# Young Australian women’s accounts of smoking and quitting: a qualitative study using visual methods

**DOI:** 10.1186/s12905-017-0500-1

**Published:** 2018-01-05

**Authors:** Zoi Triandafilidis, Jane M. Ussher, Janette Perz, Kate Huppatz

**Affiliations:** 1 Translational Health Research Institute, Western Sydney University, Locked Bag 1797, Penrith South, NSW 2751 Australia; 2 School of Social Sciences and Psychology, Western Sydney University, Locked Bag 1797, Penrith South, NSW 2751 Australia

**Keywords:** Young women, Smoking, Cessation, Qualitative, Interviews, Photography, Discourse analysis

## Abstract

**Background:**

Although the overall rate of smoking in Australia continues to decline, the rate of decline has begun to slow. Rates of smoking among young women in Australia have been a particular concern, which has led to the development of targeted public health campaigns. Poststructuralist theory has successfully been used in research to explore the way in which young women experience smoking. However, there is an absence of poststructuralist analysis of young women’s experiences of quitting. This study aims to address this gap.

**Methods:**

We carried out 27 interviews with young Australian women smokers and ex-smokers. Eighteen of those women then participated in a photography activity and follow-up interviews. A Foucauldian discourse analysis of the data was conducted.

**Results:**

Through our analysis, we identified three discourses: ‘The irresponsibility of smoking: Quitting as responsible’, ‘The difficulties of quitting: Smoking as addictive’, and ‘Making a decision to quit: Smoking as a choice’. In relation to these discourses, participants took up contradictory positions of responsibility and resistance, addiction and agency. Taking up these positions had implications for young women’s subjectivity, and the way they engaged with tobacco controls and cessation support.

**Conclusions:**

The analysis highlights the complex and contradictory nature of young women’s experiences with smoking and quitting. The study’s findings are considered in relation to the improvement of tobacco control policies and cessation support programmes targeted at young women.

## Background

Growing public awareness of the negative health implications of smoking, and the erosion of “smoking-positive cultures” have contributed to declining rates of smoking in Australia for over fifty years (pg. 25) [1]. However, recently the rate of smoking decline has slowed [2]. Considering that smoking remains one of the leading causes of preventable death and disease [3], a decrease in the rate of smoking decline is worrisome. Rates of smoking among young women are an area of particular concern. In Australia, young women are taking up smoking at a younger age than men [4]. Smoking among young women is associated with negative health outcomes, including increased mortality rates, death from cancer, circulatory, respiratory and other diseases [5]. Compared to men, women are at an increased risk for certain smoking-related diseases, due to factors such as oral contraceptive use [6]. However, if women quit smoking before the age of 30 they can avoid more than 97% of the excess mortality risk associated with continued smoking, which indicates that young women should be a target group for intervention [7].

Research on smoking cessation has primarily focused on adult smokers, which has resulted in a lack of awareness of young peoples’ experiences of quitting [8]. Young women are aware that smoking is risky, and are highly motivated to quit, but often report a sense of hopelessness in relation to the difficulty of quitting [9]. Some young women are concerned about the social, emotional, and physiological ‘costs’ of quitting smoking [10]. These costs include the loss of the perceived benefits of smoking, such as social advantages and a method of weight control [11]. It has also been reported that that young women are reluctant to engage with smoking cessation programmes, preferring to quit on their own, or with the support of a friend [12]. These studies highlight the need for further research which focuses on constructions and experiences of smoking cessation among young women, in order to better understand barriers to quitting, and to allocate tobacco control resources more appropriately. This is the aim of this paper.

Smokers’ experiences of quitting are shaped by prevalent social and cultural discourses surrounding health and wellbeing. In wealthy neoliberal western countries, a prevailing ‘healthism’ discourse maintains that the pursuit of good health is an individual’s responsibility [13]. Rational, responsible smokers are expected to maximise their health and avoid risk by complying with tobacco control policies and making attempts to quit smoking [14]. The majority of smokers appear to assume this position of responsible, health-seeking citizen, by saying they are interested in quitting [15] and making attempts to quit or cut down [16]. However, the process of quitting smoking can be difficult. Few quit attempts are successful [17], and most smokers make multiple attempts to quit before achieving long-term cessation [18]. At the same time, many smokers also challenge these health imperatives, expressing resentment and resistance towards quitting smoking [19]. By maintaining a smoking identity, smokers risk being positioned as irrational, and experiencing the negative social stigma applied to those who smoke cigarettes [20].

In the 1980s researchers began to identify nicotine and tobacco products as dependence-producing. Neuroscientists have developed a brain disease model of addiction, where nicotine is shown to activate brain pleasure centres – establishing a neurological basis to addiction [21]. Smoking dependence also relates to the context in which smoking takes place, smoking rituals, and sensory stimuli such as touch, taste and smell [22]. Characteristics of nicotine dependence include smoking soon after waking, smoking more than 10 cigarettes a day, and a history of withdrawal systems with previous quit attempts [22], while withdrawal from nicotine is associated with negative psychological and physiological effects, such as irritability, frustration, anger, anxiety, difficulty concentrating, increased appetite, restlessness, depressed mood, and insomnia [23]. These findings led to the inclusion of tobacco use disorder and tobacco withdrawal in the Diagnostic and Statistical Manual (DSM) of the American Psychiatric Association (APA), and the International Classification of Diseases (ICD) of the World Health Organisation (WHO) [24]. These physiological and psychological conceptualisations of smoking and addiction have contributed to the medicalisation and commodification of smoking cessation [25], and the development of a range of psychological interventions, such as counselling, and group interventions [26]. In Australia, health professionals advise pharmacotherapy for smoking cessation, such as nicotine replacement therapy (NRT), varenicline, and bupropion, as well as non-pharmacological supports, including group or individual counselling, cognitive and behavioural coping strategies, written information, and telephone counselling [22].

Most smokers stop smoking without formal help, either by cutting down, or quitting abruptly, also known as quitting ‘cold turkey’ [27]. However, smokers who are more nicotine-dependent are more likely to use pharmacotherapy and seek behavioural support to assist in quitting [18]. Health professionals in Australia are instructed use the 5As approach: ask patients about tobacco use, assess their willingness to quit, advise quitting, offer assistance, and arrange to follow-up with patients [22]. This approach draws on cognitive theories, such as the stages of change (SOC) model, which is based on the assumption that smokers are rational and coherent, who make gradual steps towards quitting [28]. However, these theories assume that the attitudes and beliefs shared by smokers are expressions of stable underlying cognitive structures, an assumption challenged by discourse analysists, who argue that understandings of smoking behaviour are complex and contradictory, shaped by a range of environmental and social factors [29, 30].

Counter to cognitive theories of smoking cessation, discourse analysists have sought to examine the way smokers position themselves in relation to cultural discourses of smoking and addiction. For example, adopting a subject position as an addict can provide a causal explanation for the continuation of a seemingly irrational behaviour such as smoking [13]. This subject position is enabled by tobacco control campaigns which construct smokers as powerless against their addictions, and position young adult smokers in particular, as addicts who lack self-control, discipline and willpower [19]. However, many young adults resist this construction, instead positioning themselves as ‘in control’ of their smoking, constructing their smoking as a choice rather than an addiction [31]. Young adults are often reluctant to describe themselves as having a ‘full-fledged’ addiction to smoking, and are more likely to identify with ‘weaker’ forms of dependence, such as being socially or emotionally dependent on cigarettes [30]. Contradictory discourses of smoking and quitting are also evident in in the accounts of adult smokers, who draw on notions of indulgence and control, addiction and abstinence, to account for their continued smoking and inability to quit [32, 33]. Poststructuralist theory can provide an explanatory theoretical framework for these contradictions, conceptualising smoking identities and practices as fluid and non-linear, associated with multiple, complex subject positions [34]. Poststructuralist theory allows us to recognise that young women can move between ‘smoker’ and ‘non-smoker’ identities, and that the transition from ‘smoker’ to ‘ex-smoker’ is a process of becoming rather than being [34].

Whilst poststructuralist theory has been used to inform explorations of contradictory constructions in young women’s accounts of their smoking [35, 36], these studies have not considered how young women’s constructions of smoking relate to their experiences of quitting. The present paper is necessary to improving our understandings in this area. Empirical findings from a qualitative study of young women smokers and ex-smokers are analysed using a poststructuralist approach, in order to address the following research questions: How do young women smokers and ex-smokers construct quitting and continued smoking? How do young women smokers and ex-smokers position themselves in relation to discourses of quitting and continued smoking?

## Methods

This study draws on data from a broader qualitative study which aimed to explore young women’s constructions and experiences of cigarette smoking. The study was made up of three stages. In the first stage, twenty-seven young women smokers and ex-smokers participated in exploratory semi-structured interviews. Eighteen of those women opted to participate in the second and third stages: a photography activity and follow-up interviews. The triangulation of different qualitative methods allowed us to understand young women’s experiences of quitting in a way that would not be possible through interview accounts or photographs alone. The study was carried out in metropolitan New South Wales. However, the majority of interviews (*n* = 35) were conducted over the phone, allowing us to recruit participants living interstate and regionally. The data were analysed using Foucauldian discourse analysis and positioning theory.

### Procedure

Participants were recruited through the distribution of flyers at train stations and university campuses, which asked, “Are you a young woman who currently smokes cigarettes, or an ex-smoker?” The study was also advertised on social media, with the message: “3 stage study of women’s experiences of smoking and quitting”. These two different messages were used to recruit women who might identify as a ‘smoker’ or an ‘ex-smoker’, as well as women who might not identify as ‘smokers’ or ‘ex-smokers’, but see themselves as ‘smoking’ or ‘quitting’. During the first stage of the study participants engaged in interviews which explored their experiences with smoking, quitting, and representations of smoking in the media. Eighteen of those women then chose to go on to stage two of the study, a photography activity, where they were invited to take photographs of their experiences with smoking. The process of taking photographs helps participants to reflexively engage with their smoking practices in the context of their everyday lives [37]. The photography activity drew on elements of a photovoice study, a method which actively engages participants in the research process, allowing them to share their ‘voice’ [38]. We also drew on elements of a photo-elicitation study, where the discussion of photographs in the context of an interview allows for the elicitation of rich, in-depth data [39]. This combination of photography methods has proven successful in previous qualitative studies of women’s health (see [40]). Participants took on average 3 weeks to complete the activity and submitted 157 photographs. The 18 women from stage two of the study then went on to the third stage, a follow-up interview. Following their first interview, participants were sent a copy of their interview transcript. During the follow-up interview participants were given an opportunity to reflect on their first interview, and the interviewer was able to ask follow-up questions. During the follow-up interviews, participants also discussed their photographs, and their experiences of the photography activity. The stage one and stage three interviews ranged between 25 and 90 min, and were audio recorded.

### Analysis

Poststructuralism refers to a school of thought that emerged in the 1960s and 1970s which challenged traditional understandings of language, meaning and subjectivity [41]. A poststructuralist approach considers subjectivity as multiple, and as constituted and reconstituted through language and discourse [42]. In doing poststructuralist analysis there is an opportunity to be transformative, not only through the deconstruction of current ways of knowing, but also in the development of strategies for change [43]. Foucauldian Discourse analysis was used to examine the discourses of smoking and quitting available to young women [44], and positioning theory allowed us to consider the subject positions made available through these discourses [45]. Positioning theory also provided a way of conceptualising young women as capable of taking up, resisting, and repositioning, multiple, often contradictory subject positions, such as ‘smoker’ and ‘ex-smoker’, ‘addict’ and ‘agent’.

We followed a style of Foucauldian discourse analysis outlined by Ussher and Perz (2014) [46]. The first step of the analysis involved reading transcripts and viewing the photographs. This process began when the first author integrity checked the professional transcriptions made from the interview audio recordings. The data were read and viewed again and a coding frame was developed, allowing us to code the interviews and photographs together using NVivo, a qualitative data analysis software program. This software allowed us to do an integrated analysis of the data, and identify the similarities and differences across the interviews and photographs [47]. The coded data were then summarised, and these summaries were read by all four authors, allowing us to identify dominant smoking and quitting discourses. Having identified these discursive constructions, we were then able to examine the function of these constructions, the subject positions available to participants, and implications of these positions for participants’ subjectivity and smoking practices.

The triangulation of research methods often requires the privileging or weighting of a particular data set which is better suited to the research questions [48]. Given the close relationship between discourse and verbal language, and the study’s aim to explore young women’s discursive constructions of quitting and continued smoking – the interview data were given priority during analysis. The photographs were analysed alongside the interview transcripts, and helped to provide further insight into the materiality of participants’ experiences [49]. As with their interview accounts, participants’ photographs were not considered to be a direct representation of reality, but rather, another account of their experiences of smoking and quitting produced within the context of the study [50].

## Results

The sample was made up of 27 young women aged between 18 and 31. Participants reported that they started smoking regularly between the ages of 13 and 24 years, and the average age of regular smoking was 16 years. Participants had been smoking for between two and 16 years, for an average length of 7 years. Eighteen of the participants were current smokers. Of those participants who smoked, most smoked less than 15 cigarettes a day (56%), with a smaller number smoking 15 or more cigarettes a day (22%), or not smoking on a daily basis (22%). The remaining nine participants had quit smoking at the time of recruitment. Participants’ length of cessation ranged from 2 weeks to 2 years. By including both ‘smokers’ and ‘ex-smokers’ in the sample we were able to bring together their experiences, and deconstruct binary understandings of smoking and non-smoking. Most of the sample said they were Anglo Australian (63%), and the remaining women identified as being from Aboriginal Australian, Asian, American, European, Middle Eastern, and Pacific Island cultural backgrounds. When asked about their social class background, most participants described themselves as working-class (44%) and middle-class (48%), and a smaller number identified as upper-middle class (7%). Participants mostly characterised themselves as heterosexual (67%), and bi-sexual (26%), with a smaller number identifying as lesbian (7%). At the time of the study, one participant was pregnant, and seven others had children.

During our analysis, we identified three dominant discourses in participants’ accounts of quitting: ‘The irresponsibility of smoking: Quitting as responsible’, ‘The difficulties of quitting: Smoking as addictive’, and ‘Making a decision to quit: Smoking as a choice’. Participants’ names have been replaced with pseudonyms, and their smoking status is provided in parenthesis to contextualise quotes.

### The irresponsibility of smoking: Quitting as responsible

Most participants discursively constructed smoking as irresponsible, and quitting as a way of being responsible. In positioning smoking as irresponsible, participants spoke about the impact of smoking on their health, the financial cost of their smoking, and the impact of smoking on partners, parents, friends, and children. For example, Caitlyn (smoker) said she wanted to quit smoking because, “It makes me sick, and it’s expensive.” Gemma (ex-smoker) said she quit because she was “Wanting to save money and wanting to be healthy.” Jing (ex-smoker) explained why she wanted to remain a non-smoker: “My boyfriend doesn’t want me to smoke. If I go back to smoking he will be very angry and won’t marry me.” Briana (smoker) spoke about her chances of developing a smoking-related illness, saying, “I don’t want to have to put my family through that because of a stupid decision I made.” In describing her continued smoking as a “stupid decision”, Briana implicitly positions her smoking as a choice, and herself as responsible for the negative health outcomes and impact on her family that result from her continued smoking.

**Fig. 1:**
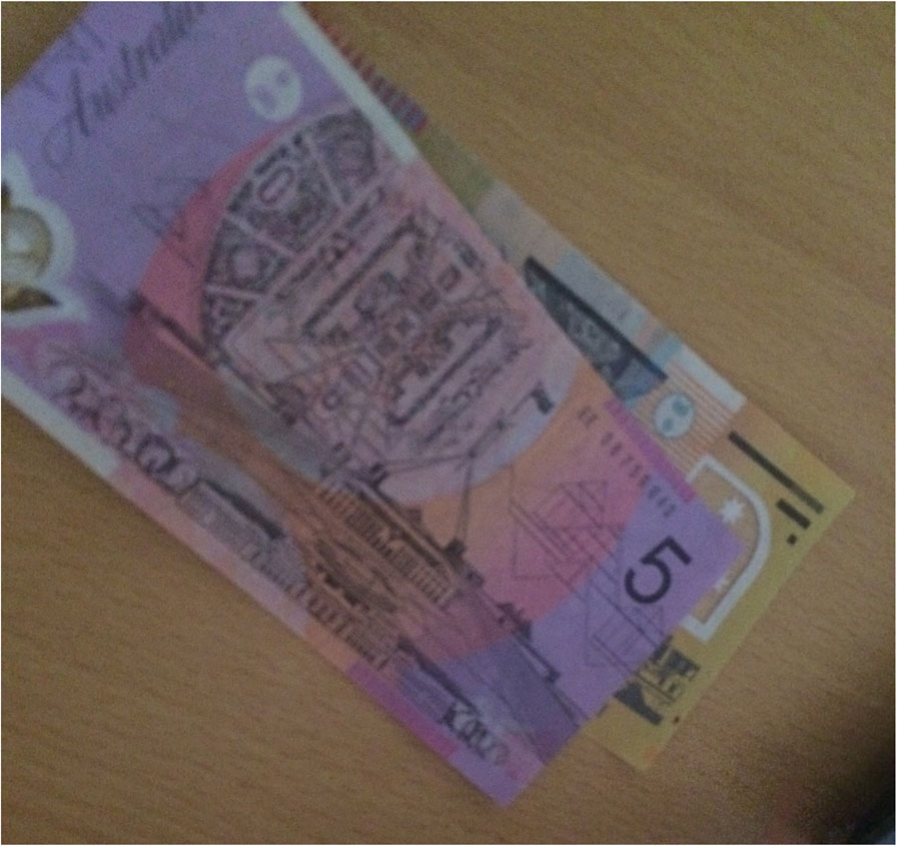
“I’ve actually got money now.” (Ashlee, ex-smoker)

Participants’ status as ‘smokers’ or ‘ex-smokers’ had implications for their positioning of self and subjectivity. For participants who had not quit, the irresponsibility associated with their continued smoking was described as having negative implications for their subjectivity. For example, Sarah (smoker) spoke about her inability to quit smoking during pregnancy, saying, “I hated myself, and I still hate myself for it today.” Conversely, participants who had quit smoking were able to take up a position of responsibility. For example, Ashlee (ex-smoker), a single mother with three children, said she took a photograph of money (Fig. 1) to represent the savings she had made since quitting smoking. Ashlee positions herself as financial responsible after quitting smoking, saying, “I’m not scraping through to get bread or milk anymore, I’ve actually got money there. And I actually have a savings account open as well now.” Lisa (smoker), reported that a past quit attempt made her feel “self-righteous”, saying, “I was somehow a better person because I was not addicted to it…I think I was just proud of myself…like ‘I’m a better person now because I’m not smoking cigarettes.” Lisa’s account highlights how adopting an ‘ex-smoker’ identity allows young women to adopt a more positive sense of self, and avoid the negative stigma of an ‘addicted smoker’ identity.

In negotiating discourses of responsibility, a number of participants took up a position of resistance in relation to quitting smoking. For example, Rachel (smoker) explained, “You get told what to do almost all the time and it's like that one small way of going against what everyone is telling you to do.” This position of resistance had implications for the way participants, such as Rachel, responded to tobacco control measures. She said,

Right before the price goes up or right before a new ad campaigns starts, I’ll see that and I’ll be like, “You know what? I'm going to keep smoking just to be stubborn.”

Although Rachel takes up a position of resistance, she also adheres to discourses of responsibility, by then saying, “I definitely intend to quit before I decide to start a family.” In a similar vein, participants spoke about intending to quit when they were older. For example, Jessica (smoker) said, “I haven’t actually really tried [to quit] because I don’t want to…I’ll try and quit when I’m 30 and see how I go.” By purporting that they “intend to quit” in the future, young women, such as Rachel and Jessica, can position their current smoking as temporary, and distance themselves from a fixed identity as a ‘smoker’.

Other participants distanced themselves from the stigma of a smoking identity, and demonstrated responsibility, by saying they would “cut back” or only smoke “a few cigarettes a day”. For example, Sarah (smoker) said, “I do want to quit, but it doesn’t bother me if I continue to have a few cigarettes a day.” Shayma (smoker), said, “I basically know that I’m just someone that probably won’t stop smoking. I’ll probably cut back.” Occasional smoking allows young women to demonstrate a sense of agency and control over their smoking. By cutting down their smoking, but not quitting, young women can simultaneously take up positions as responsible and resistant.

### The difficulties of quitting: Smoking as addictive

Accounting for the difficulty in quitting smoking, participants constructed their smoking as an addiction. For example, Hannah (smoker) said, “Quitting cigarette smoking is like trying to quit heroin. It’s similar because it’s such a strong drug in a way.” By situating smoking within a wider drug discourse, Hannah can take up a position as an addict, and can account for her continued smoking. Another participant, Danielle (smoker), said, “It’s an addiction, it’s not really a choice. I would love to stop if it was that easy, but it hasn’t seemed to be so far.” Danielle positions herself as an addict who lacks “choice”, which again, helps to account for an inability to quit smoking. This position of addiction was also assumed by Stephanie (ex-smoker), when she was trying to quit smoking:

I was always one of those people that would say, “I’m definitely not addicted, I can quit whenever I want, I just don’t want to.” And, when you do try and quit you realise you’re kind of really addicted to them.

Stephanie’s account suggests that acknowledging a lack of control over smoking, and taking up a position as an addict, may be necessary in taking up an ‘ex-smoker’ identity. However, this is not necessarily a comfortable or easy position for a smoker to find herself in. Stephanie went on to talk about the “embarrassment” she felt at being addicted to smoking, highlighting how the stigma associated with addiction can negatively impact on subjectivity.

Several participants who had quit smoking continued to position themselves in relation to discourses of addiction. For example, Gemma (ex-smoker) said,

I do miss it. If someone offered me a cigarette without any repercussions or me guaranteed not to get addicted, I’d probably take it, but, I know that if I did have another cigarette I’d start smoking again.

Stephanie (ex-smoker) offered a more fatalistic account of her addiction, saying, “If I start smoking again, I will probably continue smoking until the end. So I’m determined not to start smoking again.” These accounts highlight how young women construct quitting smoking as an on-going process, and addiction as having no fixed end point. Constructing smoking as an addiction might prevent young women who have quit smoking from resuming smoking practices. Conversely, some participants resisted being positioned as an addict. For example, Rachel (smoker) said, “I don't really have an addictive personality and I wouldn't consider myself addicted to cigarettes.” In this account, Rachel constructs addiction as being a quality of a personhood that is different to her own, which allows her to avoid the stigma associated with being positioned as an ‘addict’.

**Fig. 2:**
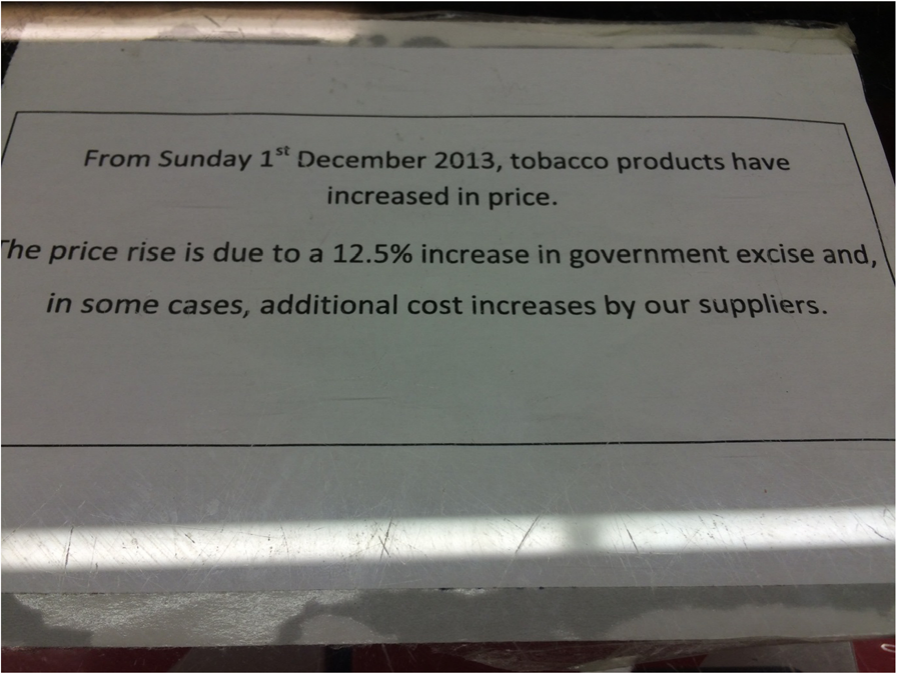
“when I saw that sign it just made me angry” (Shayma, smoker)

Addiction discourse was used in participants’ accounts of tobacco control measures, with varying effects. For example, Shayma (smoker) said she took a photograph (Fig. 2) of a sign advising of an increase in government taxation on tobacco because it made her “pissed” and “angry”. She explained,

Back then it was really cheap and people would easily get addicted because it’s very cheap and you can afford it. But now, people are finding it really hard to afford them, so it’s kind of like “I’m going to buy it anyway, but I can’t afford it. What can I do? I’m addicted to it, I need it.” I feel like it’s kind of cheating in a way…Not that I’m anyone suffering from that, but there are a lot of people that are.

Drawing on addiction discourse, Shayma is able to position taxation on tobacco as unethical and as “cheating”. Sarah (smoker) spoke about the graphic health warnings on cigarette plain packaging, saying,

It’s not going to work; it’s not going to stop people from smoking. Seeing a picture doesn’t stop your whole body, and your whole brain being tricked into, “I’m not addicted anymore.”

Sarah draws on a neurological explanation of addiction which locates the source of addiction within the body, allowing her to position external stimuli, like the graphic health warnings on cigarette packaging, as ineffective in treating her smoking addiction. Conversely, Emily (ex-smoker), said,

I didn’t like having cigarettes that had big pictures of cancer and all that sort of stuff on them. I thought it was gross and you know, “what if I looked like this”, and yeah, my brain decided it just didn’t want to smoke anymore.

Emily also draws on a neurological explanation for her smoking, but, in opposition to Sarah, positions graphic health warnings as effective in helping her “brain” to not want to smoke. Sarah and Emily’s contrasting accounts highlight how addiction discourse can have differing effects on the way young women construct the impact of tobacco control measures on their ability to quit.

#### Addiction as a social and psychological ‘habit’

**Fig. 3:**
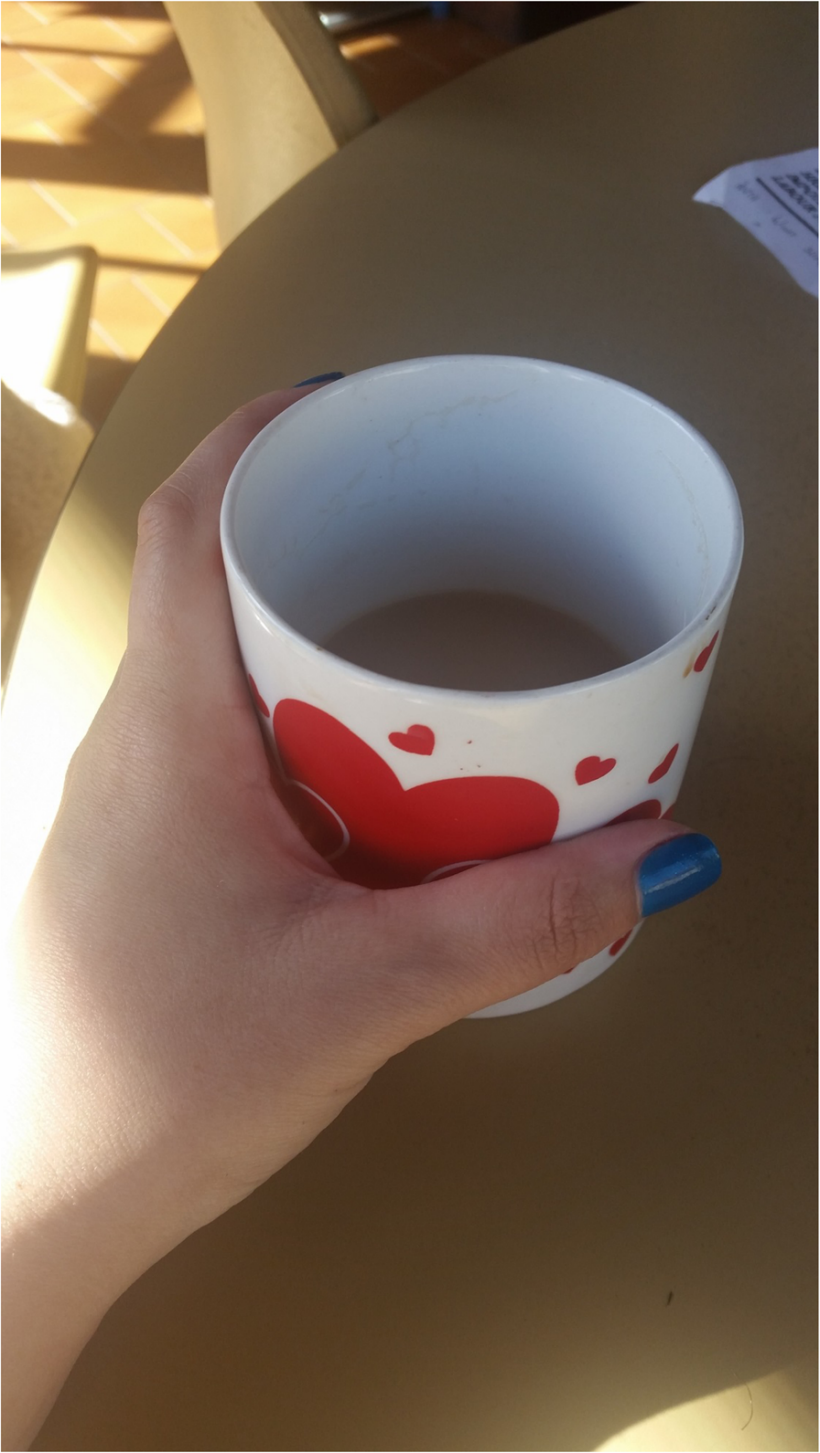
“Having a cup of tea makes me want a smoke” (Courtney, ex-smoker)

In their accounts of quitting, participants challenged the idea that their addiction was only physiological by emphasising the habitual aspects of their smoking. As Sarah (smoker) explained, “I’m addicted to the habit rather than the craving I think.” In their accounts of smoking being a habit, participants positioned their smoking as “mental”, “psychological”, and subconscious. For example, Courtney (ex-smoker) took a photograph of a cup of tea (Fig. 3), which she described as a “trigger”. She said, “If someone says, ‘Oh, do you want a coffee or a tea,’ I go, ‘Yeah.’ And, then in my head I’m like, ‘[I] should light a cigarette.’ And, then I’m like, ‘No I don’t smoke.’” In her account, Courtney positions her ability to disrupt her smoking habits as a psychological process located in her “head”. Like Courtney, many other participants spoke about how their smoking habits were paired with other habits and addictions, such as alcohol and coffee. For example, Shannon (smoker) said “it’s really hard to drink [alcohol] and not smoke”, and Tara (smoker) said “cigarettes are really good with drinks, like coffee. I find that every night before I go to sleep, I look forward to a coffee and a cigarette in the morning.”

Participants gave accounts of how the habitual nature of their addictions rendered nicotine replacement products ineffective. For example, Shannon (smoker) said, “I’m not a fan of the nicotine patches and things like that…I think a lot of the hard work in it, particularly when you’re younger, is mental and social.” Brittany (smoker), spoke about using a nicotine inhaler, saying,

I wanted a cigarette, even though I had the nicotine, I still got that kind of nice feeling of relaxation but it was the actual cigarette, like lighting it and stuff. I like the act of smoking.

In place of nicotine replacement products, participants emphasised the need for mental strength and willpower when quitting, saying “It’s just all a mental thing. It comes down to how strong your willpower is” (Tara, smoker). Willpower was said to involve “using the mind” to “move past”, “distract” or “forget about” cravings. A lack of willpower was seen by participants as being a barrier to quitting smoking. For example, Hannah (smoker) said, “I feel like I don’t have enough willpower to quit.” Another woman, Caitlyn (smoker), said, “I just need to get the willpower and give up.” In contrast to Hannah’s account, Caitlyn constructs willpower as a non-fixed quality, and something you can “get”. This construction allows young women smokers, such as Caitlyn to take up a position of agency in relation quitting smoking.

Participants spoke about the gendered nature of smoking addiction, contrasting their own habitual addictions to men’s physiological addictions. For example, Megan (smoker) spoke about how her father was “physically addicted” to smoking, while her and her mother were “situationally addicted”, and smoked in response to “environmental factors”. Another participant, Sarah (smoker), distinguished between her and her male partner’s addictions, saying, “His body is addicted to the ingredients of the smokes, like an actual cigarette, whereas I think me, personally, I’m more addicted to the five minutes of peace and relaxation.” This gendered construction of addiction had implications for how participants understood their experiences of quitting. For example, Julie (ex-smoker), spoke about her husband being much more physically addicted to cigarettes, saying, “He has smoked probably 100 thousand more smokes than what I have. I guess it is so much easier for me to let it go.” The positioning of habitual addictions as “easier” to overcome may result in women, such as Julie, experiencing greater pressure to quit, or feeling less entitled to support.

### Making a decision to quit: Smoking as a choice

In contrast to their accounts of addiction, participants constructed smoking as a choice, and emphasised the importance of making a decision to quit. For example, Megan (smoker) spoke about how being “badly addicted” to smoking made it hard to quit, but went on to say “ultimately it’s someone’s choice [whether they smoke]”. Another participant, Courtney (ex-smoker), said, “When you’re an adult you have the choice and the freedom to [smoke]…unless they want to [quit], they won’t do it.” In her account, Courtney draws on the liberal discourse of freedom to reinforce the construction of smoking as a choice, and the importance of deciding to quit. Sarah (smoker) also emphasised the importance of making a decision to quit, saying, “I think people have to want to give it up and if they don’t want to give it up…it’s not really going to happen.”

The construction of smoking as a choice, and quitting as a decision had implications for subjectivity, allowing participants to develop a sense of agency and optimism around quitting. For example, Chelsea (smoker) takes up a position of agency in relation to her smoking, saying, “If I really don’t want to [smoke], I’m sure I could stop”. This optimism is also evident in the following comment from Rachel (smoker):

As cliché as it sounds I’m sure I could quit if I really wanted to. I want to do it on my terms. I don’t want to do it as in I’m giving in to everyone constantly telling me not to smoke.

In stating that quitting needs to be on her “terms”, Rachel is positioning herself as needing to be agentic, and in control of the quitting process. Another participant, Shayma (smoker), spoke about quitting, saying, “The idea that I’ve done it once makes me feel good because I know I can do it again.” Shayma’s account illustrates how a position of agency and a sense of optimism can reinforce a positive sense of self, where Shayma can “feel good” about her past quit attempts, and motivated to make future quit attempts. Another participant, Lisa (smoker), said,

I can’t continue, and I can’t afford [cigarettes]. They’re going up at a phenomenal rate. Like for a month, I do not have enough money…to justify smoking. It’s ludicrous. So I will quit and I know that I can, it’s just a matter of actually having that little spurt to do it.

Lisa takes up a position of agency, saying she “can” and “will” quit, but indicates she requires something further to prompt her. Lisa’s account highlights that despite constructing quitting as a choice, and herself as agentic in relation to smoking, she may need further motivation or support to quit. However, in another account given by Lisa, she describes a loss of optimism and agency:

The first time [I quit] I kind of had the optimism that you know, “tomorrow I won’t be a smoker, tomorrow I won’t want a cigarette.” Whereas now I know that that doesn’t actually really go away for a very, very long time.

This account from Lisa highlights how the sense of optimism that results from the construction of smoking as a choice, may diminish with multiple quit attempts.

By constructing smoking as a choice, participants were able to dismiss tobacco control messages. Again, talking about graphic health warnings on plain cigarette packaging, Sarah (smoker) said, “I don’t think any picture will…you have to want to quit, you have to want to do it.” A similar response was given by Jessica (smoker), who said, “I don’t think the packages will have anything to do with people’s decisions…if they’re going to smoke, they’re going to smoke.” Sarah and Jessica’s accounts show that the construction of smoking as an individual choice can lead young women to be dismissive of population-level tobacco control interventions.

The construction of smoking as a choice had implications for how participants approached quitting. Participants favoured quitting ‘cold turkey’, without any assistance, as it was seen to be demonstrative of “strength”, agency and control over smoking. For example, Courtney (ex-smoker) gave the following account of quitting cold turkey,

It’s a bit empowering to turn around and say “well I didn’t use anything I just stopped”, it makes you feel good about yourself that you can just quit like that.

The positive impact of this sense of agency is also evident in Jessica’s (smoker) account,

When you use a programme or stuff like that, I feel like you haven’t got the strength to maintain it, whereas if you go cold turkey, you’ve done it all and you’ve got the strength, and yeah, it’s going to be a struggle, but you’ve achieved it, so you feel like, “wow, I did that by myself, I can do it. I don’t need help”.

**Fig. 4:**
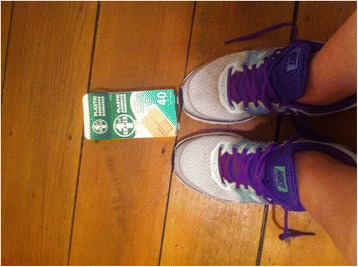
“Cigarette Wars, A New Hope.” (Megan, smoker)

Courtney and Jessica’s accounts suggest that the sense of agency gained from quitting cold turkey has positive implications for subjectivity, leading them to “feel good” about themselves. However, Courtney and Jessica’s assertions, “I didn’t use anything” and “I don’t need help”, reinforce a discourse of individualism, which may negatively impact on young women’s willingness to seek support when quitting smoking. This discourse of individualism was also evident in Megan’s (smoker) photograph of Band-Aids at her feet (Fig. 4). She explained this photograph by saying,

I put the Band-Aids on the ground to kind of push the idea that, this is something I need to heal within myself. I’m not going to get better at this unless I stop the smoking and heal the damage I’m doing to my lungs, and like stop further damage that I’m doing…there’s a hope that I can get the balls to say that I want that more than the cigarette with my wine and my beer.

In talking about her need to “heal within” herself, Megan positions herself as personally defective, and individually responsible for the damage she is doing to her body. Megan’s account of needing to “get the balls” in order to stop smoking relates to gendered discourse which positions men as having greater agency and control over their bodies.

## Discussion

Public health initiatives, such as graphic health warnings on cigarette packets, often target individual smokers, reinforcing the notion that smokers are individually knowledgeable and responsible for the risks of their smoking [51]. Participants in our study took up positions of responsibility by reiterating the irresponsibility of smoking, and expressing their desire to quit, a finding consistent with previous research [52, 53]. The cost of smoking and the impact of smoking on health were the two main reasons for wanting to quit or change their smoking behaviours, supporting previous research [16, 54]. The responsibility participants took for both their own health, and the effect of their ill health on others, could relate to the unique material and discursive pressure put on women to be primarily responsible for health and to care for others [13]. Similar findings have been identified in other research, which show that women are twice as likely as men to report feeling pressure to quit smoking, saying this pressure mainly comes from children and other family members [55]. This study also provides further evidence that tobacco control policies, such as taxes and health warnings, are successful in prompting cessation, and discouraging regular smoking among young people who are experimenting with smoking [56]. However, despite being motivated to quit, some young women require additional support with the process of cessation [57], highlighting the need for comprehensive approaches to tobacco control which incorporate both population-level interventions and additional supports targeted specifically at young women.

Young women in this study also expressed resistance towards quitting, positioning quitting or cutting down smoking as a future event. Young people often perceive few short-term negative health effects as a result of smoking, which can produce a sense of invulnerability, allowing them to delay quitting until they are older [58]. This sense of invulnerability is also affirmed by the construction of smoking as a temporary, youthful phenomenon, which young people can take up and then quit when they chose to [59]. Tobacco control programmes and policies targeted at women often focus on smoking during pregnancy or women’s roles as mothers, and “represent women as adversaries of their babies-to-be” (pg. 64) [60], which may explain the accounts of young women in this study who construct quitting as happening in the future when they plan to ‘start a family’. By positioning quitting as a future event, young women acknowledge the need to quit smoking, which allows them to maintain a position of responsibility. By cutting down their smoking and adopting an identity as a social smoker, young women are able to move between the subject positions of ‘smoker’ and ‘non-smoker’. Adopting an 'in between' identity [34], young women are able to retain the socially beneficial aspects of their smoking, while avoiding the stigma experienced by habitual smokers. This indifference towards quitting smoking represents a form of passive resistance to the imperative of health [61].

This study found that addiction discourses and a position as an addict have varying implications for young women smokers and ex-smokers. Participants in this study both adopted and resisted positions as an addict – similar to a previous study of Australian smokers [62]. Addiction discourses alleviate some responsibility for smoking behaviours, allowing young women to defend their smoking and account for the difficulties they face in quitting [29]. This finding is consistent with previous research which has found that the concept of addiction may allow women who smoke during pregnancy to maintain a ‘good mother’ identity [63]. However, a position as an addict attracts stigma, and has negative implications for young women’s subjectivity, as previous research has also identified [64]. Neurological explanations of nicotine addiction, which locate the problem of addiction within the individual as opposed to the cigarette, can also lead to feelings of fatalism, hopelessness and disempowerment [33].

Young women in this study differentiated between the physiological, and the psychological or habitual aspects of addiction, a finding consistent with previous research [29, 65]. Positioning themselves as habit-addicted, rather than nicotine-addicted, allows smokers to contextualise their smoking as a social practice that forms part of their everyday lives [62]. Young women positioned their addictions as more psychological, and men’s as more physiological, which could relate to a broader gendered discourse that constructs women as more emotional, and men as more logical [66]. This discursive positioning correlates with data that show that women are generally more sensitive than men to the non-nicotine factors that relate to smoking, such as smoking cues, and positive sensorimotor effects, leading them to have greater success with non-nicotine medications and behavioural interventions, compared to nicotine replacement therapies [67]. These findings suggest that it is important that smoking cessation programmes targeted at young women present a comprehensive view of addiction, and offer social and psychological interventions, such as counselling, alongside pharmacological treatments for nicotine addiction.

Discourses around smoking being a choice are prevalent among health care workers [68], the tobacco industry [69], in tobacco control messages targeting young people [19], and has been reported in previous studies of young smokers [70]. Therefore, it is seen to be important that smokers ‘really want to quit’ [71]. The emphasis young women place on decision making in relation to quitting smoking may lead them to think they must put serious thought and planning into quitting. This focus may prevent young women from making spontaneous quit attempts, which may be more successful than planned attempts [72]. Smoking cessation research, policy and practice has concentrated on pharmaceutical and behavioural interventions, despite most smokers reporting that unassisted quitting is their preferred method, and unassisted quitting being the method with which they have the most success [73]. The use of pharmacological cessation aids sit at odds with ideas of individual choice, willpower and control, which may explain smokers’ preference for going ‘cold turkey’ [74]. Tobacco control programmes and interventions targeted at young women need to acknowledge the value of unplanned, un-medicated cessation, but at the same time, allow space for those women who need further cessation support. As noted in previous research [75], participants in this study drew on notions of willpower to understand the control they have over smoking. Young women who position themselves as lacking willpower may be reluctant to attempt quitting, highlighting the importance of cessation support that gives young women a sense of power over their smoking. One participant constructed agency in relation to quitting as masculine, a finding which relates to research where men report greater agency over cessation [76]. These findings provide further evidence for the need to improve young women’s sense of agency and power, specifically in relation to their smoking, as well as more broadly. The use of narrative therapy has been suggested as an intervention which may help smokers to make sense of these contradictory positions of addiction and agency [62]. Through a process of externalising smokers’ unsuccessful quit attempts, and reconstructing the way they identify with smoking [77], narrative therapy may help young women, such as those who participated in this study, to develop a sense of agency and power in relation to quitting smoking. This therapy could also incorporate dual process theories of smoking cessation, which integrate contradictory notions of spontaneity and preparation in their model of successful cessation [78].

Young women’s optimism towards quitting, where they underestimated their chances of becoming addicted to smoking, and overestimated their ability to quit, has been found in previous research on young smokers [79, 80]. Optimism also plays an important role in quitting, as self-efficacy and feeling able to quit are key to successful cessation [81]. However, as findings from this study, and others have shown [80], realising the difficulty in quitting may also be an important part of the quitting process. Cessation supports and health campaigns need to foster optimism among young women smokers, by positioning quitting as an attainable goal [82]. However, they also need to be careful not to present the benefits of cessation as being immediate, or ignore the physical and psychological challenges of quitting [52]. The best approach for communicating this contradictory message of optimism and difficulty, should be the subject of further research.

### Strengths and limitations

This study is one of the first on young women’s experiences of smoking and quitting to take such an innovative theoretical and methodological approach. The use of qualitative interviews, a photography activity, and follow-up interviews allowed the researcher time to build trust with the women and actively involve them into the production and analysis of data, which helped to increase the credibility of our research [83]. The triangulation of different qualitative methods added variation to the data, which allowed for a more nuanced analysis to take place [84]. The use of discourse analysis and poststructuralist theory allowed us to examine young women’s adoption of dominant discourses of smoking and quitting, as well as their resistance of them [42]. However, the triangulation of interview and photography data also presents several challenges. For instance, decisions relating to the privileging of one data set above another is a challenging aspect of the triangulation process [48]. The prioritisation of the interview data in this analysis lead to a more limited consideration of the photographs. This limitation could be addressed in future research which places more emphasis on visual analyses of young women’s experiences of smoking and quitting.

The nature of qualitative research means that findings are not generalisable to a wider population, but findings can be transferred to other settings [85]. The transferability of findings to young women of different cultural backgrounds may be limited due to the large proportion of Anglo-Australian women in the sample. Passey, Gale, and Sanson-Fisher’s (2011) research has highlighted the importance of socio-cultural context in understanding experiences of smoking among Aboriginal women in Australia [86]. Future research could look more closely at culture and ethnicity, and how these identities might shape young women’s constructions and experiences of quitting, in order to develop interventions which are specific to culturally-diverse young women. The findings of the study are also limited to the majority heterosexual sample of women. Bi-sexual and lesbian women have expressed interest in tailored smoking cessation interventions [87], and such interventions have begun to be developed in Australia [88]. Further qualitative research on young bi-sexual and lesbian women’s experiences of smoking and quitting could support the continued development and evaluation of such interventions. Limitations also surround the applicability of these findings to young women who are pregnant. Previous research has shown that pregnant and non-pregnant women have distinct experiences of smoking cessation [89]. Although this study included women’s experiences of smoking and quitting during pregnancy, these women made up less than a third of the original sample, which limits the transferability of our findings to this group.

## Conclusions

The findings from this study add new insight into the complexity and contradiction of young women’s quitting experiences. These findings align with the contradictory depictions of smokers inherent in tobacco control and tobacco industry agendas. For instance, while smoking is portrayed in tobacco control campaigns as a dangerous, risky and addictive habit, cigarettes have also been advertised as a source of pleasure and enjoyment, and a form of emancipation for women [90]. Findings from this study are relevant to the development of individualised interventions, and important to the evaluation of current public health approaches to smoking. The complexity of these issues calls for a comprehensive approach to young women’s smoking, where population-level policies, such as taxation, are delivered alongside local interventions targeted specifically at young women [8]. Interventions targeted at young women need to offer flexibility and variety, allowing women to take control and make their own decisions regarding cessation [91, 92].

Tobacco control messages need to challenge existing public health discourse that suggests smokers are solely responsible for their health, or have complete control over their smoking behaviours. However, it is also important that these messages do not also remove young women’s sense of agency, as this study has shown that a lack of agency in quitting can result in experiences of disempowerment and/or an inclination to resist quitting. It is imperative that programmes and policies targeting young women seek to intervene earlier than pregnancy and motherhood, as many women in Australia are now delaying having children until their 30s [93]. The focus of these programmes and policies could be directed towards improving young women’s mental health, given their reports of increasing levels of psychological distress [94], and the bi-directional relationship between smoking and poor mental health among young women [95]. As previous researchers have argued [96], it is our recommendation that in order to impact cessation rates among young women, we must improve the social and economic environment in which they are situated. The similarity in accounts of young women ‘smokers’ and ‘ex-smokers’ in this study, affirms the need for tobacco control responses which conceptualise smoking and non-smoking as non-binary, non-linear practices, and quitting smoking as being more of a process of recovery and relapse [32, 34].
